# Enhancing Stability and Capacity in Planar Zn‐Ion Micro‐Batteries via 3D Porous Ni Anode Integration

**DOI:** 10.1002/smtd.202501194

**Published:** 2025-09-03

**Authors:** Yijia Zhu, Xiaopeng Liu, Nibagani Naresh, Jingli Luo, Xueqing Hu, Sijin Liu, Georgios Nikiforidis, Mingqing Wang, Buddha Deka Boruah

**Affiliations:** ^1^ Institute for Materials Discovery University College London London WC1E 7JE UK

**Keywords:** 3D porous nickel scaffold, on‐chip energy storage, planar micro‐batteries, stable anodes, zinc‐ion batteries

## Abstract

The development of planar on‐chip micro‐batteries with high‐capacity electrodes and environmentally friendly and stable architectures is critical for powering the next generation of miniaturized system‐on‐chip smart devices. However, realizing highly stable micro‐batteries remains a major challenge due to complex fabrication processes, electrode degradation during cycling, and the uncontrolled growth of dendrites in metal‐based anodes within the confined spaces between electrodes. To address these issues, this study presents an approach that incorporates a 3D porous nickel (Ni) scaffold at the metal anode, offering improved micro‐anode stability compared to conventional planar zinc and 3D porous zinc (Zn) scaffolds. Integrated into a planar configuration with a polyaniline (PANI) cathode and a zinc‐loaded 3D porous Ni scaffold anode, this design significantly enhances long‐term cycling stability, lowers charge transfer resistance, and increases charge storage capacity from 10 to 14 µAh cm^−2^ at 0.1 mA cm^−2^ compared to the same materials deposited on traditional planar gold microelectrodes. As a result, the Zn‐ion micro‐batteries achieve notable peak areal energy and power densities of 17.22 µWh cm^−2^ and 6.98 mW cm^−2^, respectively. This work provides an effective strategy for improving the electrochemical performance and durability of planar micro‐batteries, marking a significant advancement toward the future of portable microelectronic devices.

## Graphical abstract

A 3D porous nickel scaffold loaded with zinc, integrated into planar Zn‐ion micro‐batteries with a polyaniline cathode, significantly enhances anode stability and boosts electrochemical performance. This design achieves improved cycling stability, reduced charge transfer resistance, and higher areal capacity, energy and power for next‐generation microelectronics devices.

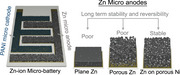

## 1 Introduction

The rapid growth of the Internet of Things (IoT) has placed miniaturization and integration at the forefront of microelectronics innovation. Remarkable advancements have been made in developing compact systems such as micro‐robots and micro‐sensors, which are poised to play key roles in a wide range of everyday applications. These ultra‐small devices ‐ often occupying less than a few cubic millimeters ‐ are highly capable in data processing and wireless communication, making them ideal for use in areas like health monitoring, medical diagnostics, and targeted disease treatment.^[^ ^1^ ^]^ However, their efficient and autonomous operation hinges on the availability of reliable and efficient power sources. As wearable and implantable microelectronics continue to shrink in size while increasing in functionality, the need for high‐performance micro‐power sources, such as micro‐batteries, has become more urgent.^[^ ^2^, ^3^ ^]^ Traditional micro‐batteries, typically based on stacked electrode‐separator configurations, face several limitations in meeting the power demands of next‐generation devices. In response, planar micro‐batteries with integrated electrode patterns have emerged as a promising alternative.^[^ ^4^, ^5^, ^6^ ^]^ Their design allows for direct integration with system‐on‐chip architectures, enables separator‐free operation, and aligns well with standard 2D microfabrication processes. Thanks to these inherent advantages, planar micro‐batteries stand out as strong candidates for powering the next generation of miniaturized smart electronics. While the reduced electrode spacing in planar micro‐batteries enhances energy density by shortening ion diffusion pathways, it also increases the risk of short‐circuiting due to dendrite growth between the electrodes ‐ particularly in systems using metal‐based anodes, such as Zn‐ion micro‐batteries.^[^ ^7^, ^8^, ^9^, ^10^ ^]^ Furthermore, in Zn‐ion batteries, the reversible stripping and plating of Zn onto the micro‐anode current collector must be well‐controlled. Unlike coin‐cell configurations that use bulk Zn foil as the anode, micro‐batteries are constrained by limited Zn mass loading and device footprint, making efficient and stable Zn cycling critical for performance and longevity.^[^ ^11^, ^12^ ^]^ In coin cells, dendrite growth on the Zn anode can be mitigated by applying artificial protective layers on the anode, designing electrolytes that promote in situ formation of solid electrolyte interphases (SEI), or optimizing the separator to physically suppress dendrites.^[^ ^13^, ^14^ ^]^ However, controlling dendrite formation in microbatteries remains more challenging due to the limited footprint of the Zn anode and the commonly adopted separator‐free design. A promising approach to address these challenges involves constructing a 3D porous structure on the anode side. This design helps maintain electrode geometry while offering multiple advantages:^[^ ^15^ ^]^ it redirects dendrite growth away from the planar electrode edges into the interior of the porous structure, thereby reducing short‐circuit risks. Additionally, the 3D architecture increases the available surface area, allowing for higher Zn mass loading and improved electrochemical kinetics. For example, Shaohong Shi^[^ ^16^ ^]^ developed a 3D Cu structure as the current collector and the battery could cycle for 700 times under 4 mA cm^−2^. As a result, the micro‐batteries can operate more effectively under high scan rates and current densities, significantly broadening their application potential in advanced miniaturized electronics.

In this study, we explored various micro‐anode configurations for Zn‐ion micro‐batteries, including planar Zn anodes, 3D porous Zn anodes, and 3D porous Ni scaffolds subsequently loaded with Zn, to evaluate and enhance anode stability while mitigating dendrite growth. The primary goal was to regulate reversible Zn^2^⁺ plating/stripping and to direct dendrite growth away from critical regions, thereby reducing the risk of short circuits. Our in‐depth analysis demonstrated that the 3D porous Ni scaffold significantly outperformed both planar and 3D porous Zn anodes in managing reversible Zn^2^⁺ plating/stripping. Moreover, Ni scaffolds of varying thicknesses demonstrated significantly improved long‐term stability during extended charge‐discharge cycling. As expected, Zn‐ion micro‐batteries employing Ni scaffold‐based micro‐anodes exhibited superior electrochemical performance ‐ delivering enhanced cycling stability and rate capability ‐ compared to conventional PANI and Zn loaded onto gold interdigitated electrodes configurations. Notably, these optimized devices achieved an impressive areal energy of 17.22 µWh cm^−^^2^ and a peak areal power of 6.98 mW cm^−^^2^. Overall, this work demonstrates a promising strategy to effectively suppress dendrite formation in Zn‐ion micro‐batteries, paving the way for the development of high‐performance and reliable planar micro‐power sources.

## 2 Results and Discussion

**Figure 1:**
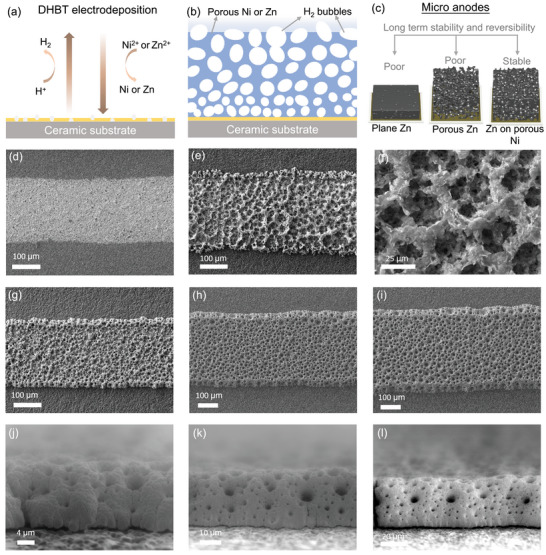
a,b) Schematic illustrations of the DHBT electrodeposition process and the resulting porous structures. c) Schematic representation of different 3D electrode architectures: planar Zn, porous Zn, and Zn deposited on a porous Ni scaffold (NZn). SEM top‐view images of d) planar Au electrode, e,f) 3D porous Zn electrode at low and high magnifications, respectively. SEM top‐view images of 3D porous Ni scaffolds deposited for g) 20 s, h) 40 s, and i) 60 s. SEM side‐view images of 3D porous Ni scaffolds after j) 20 s, k) 40 s, and l) 60 s of deposition, respectively.

The dynamic hydrogen bubble template (DHBT) technique was employed to fabricate the 3D porous structures. This method provides a template‐free, cost‐effective, and environmentally friendly approach, enabling dynamic pore formation during electrodeposition while offering material versatility and scalability for large‐scale manufacturing. As illustrated in **Figure** **1**a,b, hydrogen bubbles are generated during the electrodeposition process, creating a porous network as the material deposits around them. Subsequent studies involved depositing Zn onto planar Au micro‐electrodes, fabricating porous Zn micro‐anodes, and loading Zn onto 3D porous micro‐electrodes (Figure 1c), followed by evaluations of their stability in Zn‐ion micro‐batteries. Compared to the planar Au electrodes (Figure 1d), the 3D porous Zn micro‐anodes (Figure 1e,f) and 3D porous Ni structures (Figure 1g–i) demonstrate highly porous morphologies achieved via DHBT by varying the deposition times (20, 40, and 60 s). Figure 1j–l further presents side‐view images of the 3D porous Ni structures, clearly revealing the interconnected porous architecture.

**Figure 2:**
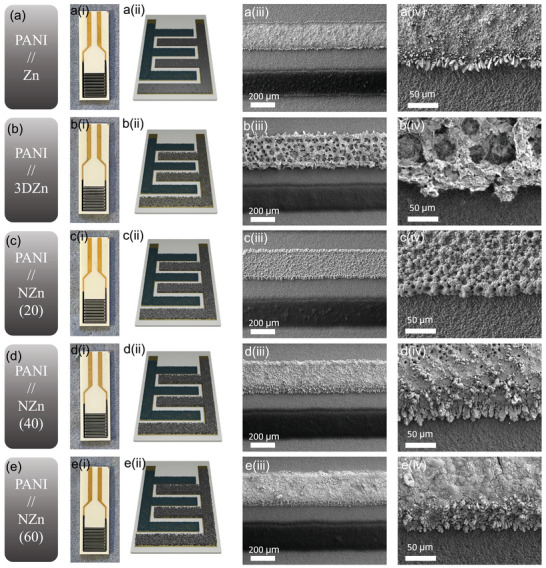
i) Digital photographs, ii) schematic illustrations, and iii, iv) SEM images at varying magnifications of a) PANI//Zn, b) PANI//3D Zn, c) PANI//NZn(20), d) PANI//NZn(40), and e) PANI//NZn(60) micro‐battery electrodes.

To gain deeper insights into the morphological evolution of the micro‐electrodes, we performed a detailed SEM investigation at various magnifications. **Figure** **2** presents the digital photographs, schematic representations, and SEM images of (a) PANI//Zn, (b) PANI//3D Zn, (c) PANI//NZn (20) ‐ corresponding to 20 s of 3D porous Ni scaffold deposition, (d) PANI//NZn (40) ‐ 40 s deposition, and (e) PANI//NZn (60) ‐ 60 s deposition. The SEM images clearly reveal that in the PANI//Zn micro‐battery configuration, small Zn crystals are observed growing at the edges of the planar Zn anode, indicating non‐uniform deposition and potential hotspots for dendrite formation. In contrast, the 3D architectures ‐ including the PANI//3D Zn and PANI//NZn devices ‐ exhibit significantly smoother and more uniform electrode surfaces. The edges of the 3D Zn and NZn anodes are notably free of protruding crystals, suggesting that the incorporation of the 3D porous structures effectively suppresses localized Zn accumulation. This uniform morphology is expected to enhance electrochemical stability, suppress dendrite growth, and improve the overall cycling performance of the Zn‐ion micro‐batteries.

**Figure 3:**
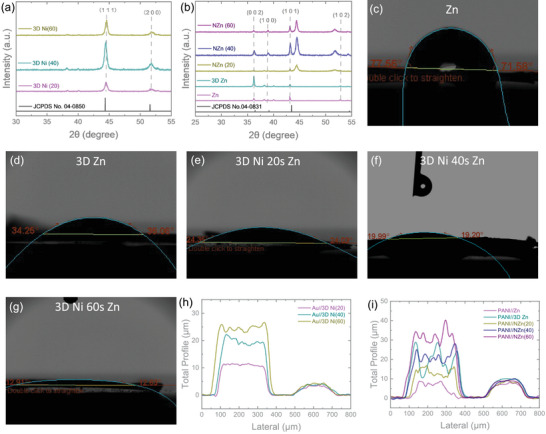
a) XRD patterns of Ni XRD standard card and 3D Ni scaffolds deposited for 20, 40, and 60 s. b) XRD patterns of Zn XRD standard card, Zn, 3D Zn, and Zn deposited on 3D Ni scaffolds (NZn(20), NZn(40), NZn(60)). Contact angle measurements of c) planar Zn, d) 3D Zn, and e–g) NZn anodes with varying Ni scaffold deposition times (20, 40, and 60 s, respectively). h) Profilometry line scans of 3D Ni scaffolds deposited on planar Au substrates. i) Thickness profiles of micro‐battery electrodes: PANI//Zn, PANI//3D Zn, and PANI//NZn(20), NZn(40), and NZn(60).

**Figure** **3**a,b presents the XRD patterns of the samples and the standard XRD card to confirm the successful formation of the target materials. The Ni scaffolds exhibit characteristic diffraction peaks at 44.5° and 51.9°, corresponding to the (111) and (200) crystal planes, respectively.^[^ ^17^ ^]^ Although the 3D porous Zn anodes were fabricated differently from conventional Zn anodes, the diffraction peaks at 36.3°, 38.9°, 43.1°, and 52.9°, assigned to the (002), (100), (101), and (102) planes, respectively,^[^ ^18^ ^]^ confirm that the deposited material is indeed Zn. Furthermore, Zn peaks were also observed when Zn was deposited onto the Ni scaffold, confirming successful loading. To assess the effect of the 3D porous structure on surface wettability, contact angle measurements were conducted. As shown in Figure 3c, the contact angle for the flat electrode without a 3D structure was ≈70°. In contrast, the 3D Zn structure significantly reduced the contact angle to 35° (Figure 3d). Even greater reductions were observed with the 3D Ni scaffolds, where the contact angles decreased progressively from 24° to 19° and 13° with increasing scaffold thickness (Figure 3e–g). These results clearly demonstrate that the introduction of 3D structures notably enhances the hydrophilicity of the electrodes. The scaffold thicknesses were further analysed and are shown in Figure 3h,i. The planar Au electrodes exhibited a thickness of ≈4.5 µm. In comparison, the 3D Ni scaffolds formed over deposition times of 20, 40, and 60 s exhibited thicknesses of ≈8, 14, and 20 µm, respectively. After deposition of the active materials, the thicknesses of the electrodes were measured. The PANI cathode layer was ≈5.5 µm thick. For the anodes, the planar Zn layer measured ≈4.5 µm, whereas the 3D Zn layer reached ≈15.5 µm. When Zn was deposited onto the 3D Ni scaffolds, the final thicknesses of the electrodes increased to ≈15, 23, and 32 µm, respectively, demonstrating that Zn deposition contributed an additional thickness of roughly 3–5 µm.

**Figure 4:**
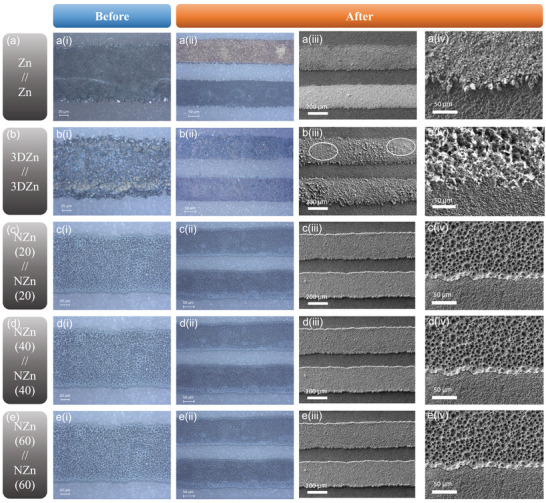
(i, ii) Optical microscope images and (iii, iv) SEM images of symmetrical cell electrodes: a) Zn, b) 3D Zn, c) NZn(20), d) NZn(40), and e) NZn(60), captured before i) and after ii–iv) 1 h discharge.

To assess the morphological stability of the micro‐anodes, we constructed symmetric cells and monitored the reversibility of Zn deposition using optical microscopy and SEM (**Figure** **4**). In conventional Zn symmetric cells with Zn deposited on planar Au integrated electrodes (IDEs), the Zn at the anode side was entirely consumed after discharge, as shown in Figure 4a‐ii–iv. In contrast, in 3D Zn symmetric cells (porous Zn deposited on Au IDEs), partial structural degradation of the 3D porous Zn framework was observed after 1 h of discharge, particularly within the circled region in Figure 4b‐iii. Moreover, the structure was not fully restored even after a subsequent 1 h charging step (Figure S1, Supporting Information), indicating limited structural reversibility of the 3D Zn anode under cycling. In planar configurations, zinc deposition was predominantly concentrated at the electrode edges in Zn symmetric cells, promoting dendrite formation. In contrast, the 3D Zn configuration effectively suppressed such edge‐localized dendritic growth. In NZn symmetric cells (Zn deposited on 3D Ni scaffolds), Zn remained intact post‐discharge due to the enhanced deposition capacity offered by the 3D scaffold (Figure 4c–e). Notably, unlike the directional Zn growth observed at the edges of planar IDEs, Zn growth within NZn symmetric cells occurred more uniformly in all directions throughout the porous structure. This spatial distribution mitigates localized dendrite formation and promotes electrochemical stability. Minor morphological differences were noted among NZn electrodes with varying scaffold thicknesses. With increasing Zn loading, some edge Zn growth was visible, particularly in NZn(60) cells, though the extent remained significantly less severe than that observed in planar IDEs (Figure 4e‐iv). These findings highlight the effectiveness of 3D porous scaffolds in enhancing the morphological robustness and mitigating dendrite formation in Zn‐based micro‐batteries. The symmetric cell results further demonstrate improved stability and lower overpotential in NZn//NZn cells compared to Zn//Zn cells (Figure S2, Supporting Information). Specifically, the NZn cells sustained cycling at 30 µA cm^−2^ for over 1000 h, whereas the Zn//Zn cells failed after ≈800 h due to short‐circuiting.

**Figure 5:**
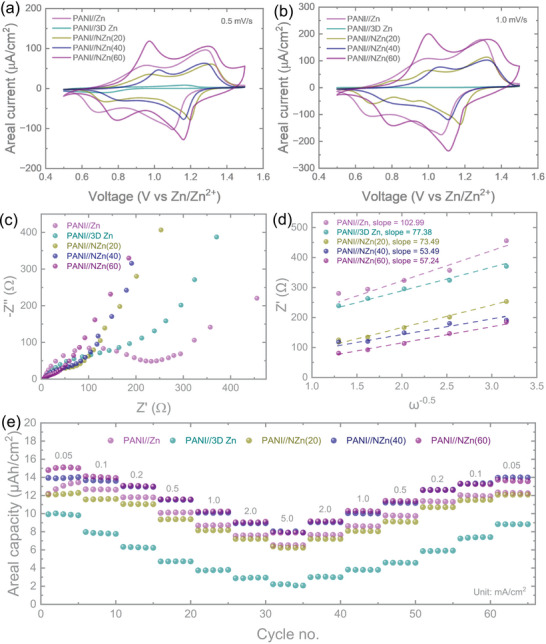
a,b) Cyclic voltammetry (CV) curves of PANI//Zn, PANI//3D Zn, PANI//NZn(20), PANI//NZn(40), and PANI//NZn(60) micro‐batteries recorded at scan rates of 0.5 mV s^−1^ and 1.0 mV s^−1^, respectively. c) Nyquist plots from electrochemical impedance spectroscopy (EIS) showing the impedance spectra of the corresponding micro‐batteries. d) Relative Zn^2^⁺ ion diffusion coefficients derived from the Warburg region of the EIS data for the five configurations. e) Rate performance of the micro‐batteries at increasing areal current densities of 0.05, 0.1, 0.2, 0.5, 1, 2, and 5 mA cm^−2^.

The electrochemical performance of the micro‐batteries was evaluated using a 3 M Zn(CF_3_SO_3_)_2_ in PVA gel electrolyte. **Figure** **5**a,b shows the comparative cyclic voltammetry (CV) curves of PANI//Zn, PANI//3D Zn, PANI//NZn(20), PANI//NZn(40), and PANI//NZn(60) at scan rates of 0.5 and 1.0 mV s^−1^. All devices exhibit two pairs of oxidation and reduction peaks, characteristic of the multiple redox processes in PANI. Additional CV curves at scan rates ranging from 0.2 to 1.0 mV s^−1^ are provided in the Supporting Information (Figure S3a–e, Supporting Information). The CV profiles of PANI//Zn and PANI//NZn devices retain similar shapes with minimal peak shifts as the scan rate increases. In contrast, the PANI//3D Zn device exhibits a notable decline in capacity at higher scan rates (e.g., 1.0 mV s^−1^), which may be attributed to the morphological instability of the porous 3D Zn anode. There are two sets of redox peaks appearing in the CV curves, located at 0.7–0.9 and 1.1–1.3 V. The two redox reactions correspond to the two‐step redox reaction of PANI, including from the fully reduced leucoemeraldine to the half‐oxidized emeraldine state (0.7–0.9 V), and from emeraldine to the fully oxidized pernigraniline state (1.1–1.3 V). In the PANI//NZn(20) microbatteries, a third set of peaks appeared at the scan rate of 1.0 mV s^−1^. As observed in Figures 2c and 4c, the Zn layer in the PANI//NZn(20) micro‐battery does not fully cover the Ni scaffold. As a result, exposed regions of the Ni surface may interact with the electrolyte, leading to undesired side reactions and the appearance of an additional redox couple. This issue is effectively resolved in the PANI//NZn(40) and PANI//NZn(60) devices, where the increased Zn loading ensures full coverage of the Ni scaffold, thereby eliminating the third redox peak. The incorporation of 3D Zn on a Ni scaffold significantly reduces the overpotential associated with both major and minor redox peaks. Overpotential, defined as the deviation between the actual electrode potential and the thermodynamic equilibrium potential (Δ*E* = *E_actual_* − *E_eq_*) serves as a measure of electrochemical reaction efficiency. In a reversible system, E_eq_ can be approximated as the midpoint between anodic (E_pa_) and cathodic (E_pc_) peak potentials: $E_{eq}\approx \frac{E_{pa}+E_{pc}}{2}$.^[^ ^19^, ^20^ ^]^ At a scan rate of 0.5 mV s^−1^, the PANI//Zn micro‐battery exhibits major and minor overpotentials of 0.13 and 0.10 V, respectively, while the PANI//NZn variants show reduced values of less than 0.10 and 0.08 V, highlighting the beneficial impact of the 3D scaffold structure on charge‐transfer kinetics. To better understand the charge storage kinetics, we analysed the relative contributions of diffusion‐controlled and capacitive‐controlled processes. Capacitive charge storage, characterized by rapid reversibility, is especially beneficial for achieving high‐rate battery performance. The relationship between peak current (*i_p_*) and scan rate (*v*) in cyclic voltammetry follows the power law: *i_p_* = *av^b^*, where *a* and *b* are adjustable parameters.^[^ ^21^ ^]^ A *b*‐value of 0.5 indicates a diffusion‐dominated process, while a value of 1 suggests a capacitive‐controlled mechanism. As shown in Figure S4a,b (Supporting Information), the calculated *b*‐values for the anodic peaks of PANI//Zn, PANI//NZn(20), PANI//NZn(40), and PANI//NZn(60) micro‐batteries are 0.98, 0.96, 0.84, and 0.88, respectively ‐ highlighting a dominant capacitive contribution. The corresponding cathodic peak *b* values are 0.78, 0.85, 0.84, and 0.87, indicating that reduction processes also gain capacitive character, particularly with the introduction of 3D Ni scaffolds. The b‐values for the minor peaks, presented in Figure S4a–d (Supporting Information), follow a similar trend, further confirming the enhanced capacitive behavior introduced by the scaffold structure.

Galvanostatic charge‐discharge (GCD) measurements were conducted for the micro‐batteries across a range of areal current densities (50 µA cm^−2^ to 5 mA cm^−2^) within a voltage window of 0.5 to 1.5 V, as shown in the Supporting Information (Figures S5 and S6, Supporting Information). While the incorporation of 3D structures at the anode did not significantly increase the areal capacities at low areal currents (which remained between 10 and 15 µAh cm^−2^ at 50 µA cm^−2^), the benefits of the engineered structure were clearly observed in rate performance. As illustrated in Figure 5e, PANI//Zn micro‐batteries delivered 13.46 µAh cm^−2^ at 0.05 mA cm^−2^ and retained 91.04% of this capacity after returning to the same current following high‐rate cycling. In contrast, PANI//3D Zn micro‐batteries exhibited lower areal capacities and reversibility (88.04%), likely due to the structural instability of the porous Zn anode discussed previously (Figure 4). Notably, devices incorporating 3D Ni scaffolds demonstrated improved rate performance and excellent capacity retention. The capacity recovery values for PANI//NZn(20), PANI//NZn(40), and PANI//NZn(60) were 98.44%, 99.72%, and 91.53%, respectively, confirming the enhanced electrochemical stability imparted by the 3D scaffold architecture. We also performed Electrochemical Impedance Spectroscopy (EIS) measurements over a frequency range of 10 mHz–100 kHz with a 10 mV voltage amplitude to further explore the charge transfer kinetics and assess the impact of incorporating 3D porous structures at the anode. The Nyquist plots (Figure 5c) reveal that the charge transfer resistance of PANI//Zn micro‐batteries is ≈220 Ω. This resistance is reduced to ≈160 Ω in PANI//3D Zn micro‐batteries, indicating moderate improvement. Notably, micro‐batteries utilizing 3D Ni scaffolds at the anode exhibit significantly lower resistances: ≈80 Ω for 20 s Ni, 60 Ω for 40 s Ni, and 40 Ω for 60 s Ni. These results clearly demonstrate that the introduction of 3D porous structures effectively minimizes internal resistance and enhances charge transfer efficiency within the micro‐battery systems. To further investigate ion transport characteristics, we evaluated the relative Zn^2^⁺ ion diffusion coefficients using the linear region of the Nyquist plots, based on the following relations: *Z*′ = (*R_L_* + *R_D_*) + σω^−0.5^ and $D_{Zn^{2+}}=\frac{R^{2}T^{2}}{2A^{2}n^{4}F^{4}C^{2}\sigma^{2}}=\frac{K}{\sigma^{2}}$; where (*R_L_* + *R_D_*) is the overall resistance (solution and charge transfer resistance), σ is the Warburg coefficient, ω is the angular frequency, *Z*′ is the real impedance component of the Nyquist plot, $D_{Zn^{2+}}$ is the Zn^2^⁺ ion diffusion coefficient (cm^2^ s^−1^), R is the molar gas constant (8.314 J K^−1^ mol^−1^), T is the cell testing temperature in Kelvin (298.13 K), 𝑛 is the number of electrons transferred per electrolyte per monomer unit of PANI, *A* is the active electrode area (cm^2^), 𝐹 is Faraday's constant (96 485.3383 C mol^−1^), and 𝐶 is the Zn^2^⁺ ion molar concentration used in the electrolyte.^[^ ^22^ ^]^ Since the electrode area and electrolyte concentrations during testing are the same, we can consider $k=\frac{R^{2}T^{2}}{2A^{2}n^{4}F^{4}C^{2}}$ the same for all the micro‐batteries. Therefore, Zn^2^⁺ ion diffusivity is inversely proportional to σ^2^, and lower slopes in the *Z*′ versus ω^−0.5^ plots (Figure 5d) correspond to higher diffusion coefficients. Among all configurations, the PANI//NZn(40) device exhibits the lowest slope, confirming superior ion transport, likely due to the optimal balance of porosity and structure in the 40 s Ni scaffold.

**Figure 6:**
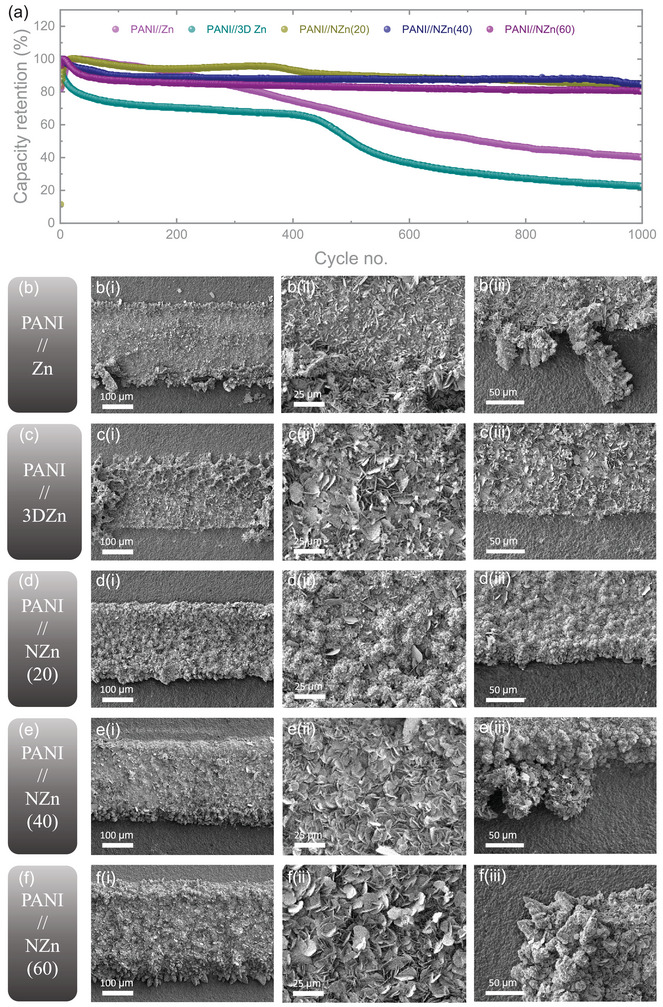
a) Long‐term cycling performance of the micro‐batteries at an areal current of 1 mA cm^−2^. SEM images of the anodes for (a) PANI//Zn, b) PANI//3D Zn, c) PANI//NZn(20), d) PANI//NZn(40), and e) PANI//NZn(60) micro‐batteries after 1000 cycles, shown from low i) to high iii) magnifications.

To further evaluate the cycling stability of the micro‐batteries, extended cycling tests were carried out at an areal current of 1.0 mA cm^−2^, as shown in **Figure** **6**a. The PANI//Zn micro‐batteries exhibited continuous capacity degradation throughout the test, retaining only ≈40% of their initial capacity after 1000 cycles. In contrast, PANI//3D Zn micro‐batteries maintained a stable capacity plateau for the first 400 cycles but experienced a notable decline thereafter. This decline is attributed to the gradual collapse of the porous 3D Zn structure, which lacks mechanical support, a hypothesis confirmed by post‐cycling SEM imaging. In comparison, micro‐batteries incorporating a 3D Ni scaffold demonstrated significantly improved cycling performance, maintaining over 80% of their initial capacity after 1000 cycles. Specifically, PANI//NZn(20), PANI//NZn(40), and PANI//NZn(60) retained 85.11%, 84.71%, and 80.35% of their initial capacities, respectively. To further examine the long‐term morphological changes, post‐mortem SEM analyses were conducted on the electrodes after 1000 cycles (Figure 6b–f; Figure S7, Supporting Information). PANI//Zn cells exhibited severe dendrite growth, particularly at the electrode edges, due to uneven Zn deposition. Although dendrites were absent in PANI//3D Zn cells, partial loss of the Zn anode and its porous structure was observed after prolonged cycling. To investigate this transition, additional batteries were tested for 500 cycles ‐ the point at which capacity fading begins. As shown in Figure S8 (Supporting Information), a majority of the 3D porous structure remained after 500 cycles, albeit with noticeable signs of degradation, further confirming the lack of structural stability in the absence of a scaffold. On the other hand, electrodes with a 3D Ni scaffold maintained their structural integrity even after 1000 cycles. Most Zn deposition was confined to the top surfaces rather than the edges (Figures 6d‐ii and 6f‐ii), effectively mitigating dendrite growth. However, with increasing Ni deposition time (from 20 to 60 s), a slight emergence of dendrites at the edges was observed, particularly in PANI//NZn(60), likely due to excessive Zn loading within the thicker scaffold. Despite this, the extent of dendrite formation in NZn(40) and NZn(60) remained substantially lower than that in PANI//Zn, highlighting the 3D Ni scaffold's effectiveness in suppressing dendrite growth ‐ especially when Zn deposition is well controlled. What's more, EDS was also done to explore the stability of the 3D Ni scaffolds. As shown in Figure S9 (Supporting Information), it's obvious that the Ni can be highly detected after cycling. With the increasing thickness of the Ni scaffold, the signal was reduced due to the thicker layer of the Zn electrode.

**Figure 7:**
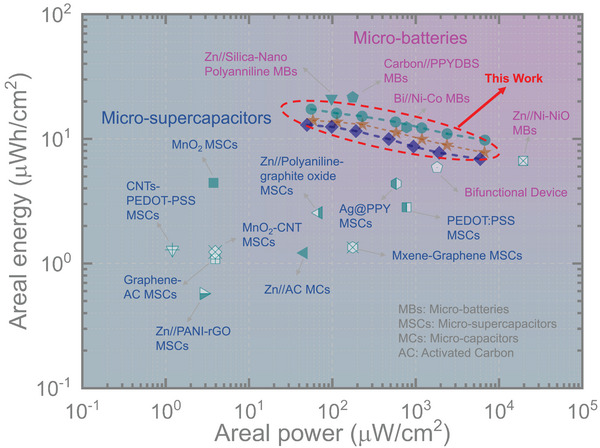
Ragone plot comparing the energy and power performance of the PANI//NZn micro‐batteries with previously reported micro‐batteries: Zn//Silica‐Nano Polyaniline,^[^ ^25^ ^]^ Carbon//PPYDBS,^[^ ^35^ ^]^ Bi//Ni‐Co,^[^ ^33^ ^]^ Zn//Ni‐NiO;^[^ ^34^ ^]^ micro‐supercapacitors: MnO_2_‐CNTs,^[^ ^31^ ^]^ Graphene/CNT/cross‐linked PH1000 film(GCP),^[^ ^32^ ^]^ Zn//Polyaniline‐graphite oxide,^[^ ^24^ ^]^ Ag@PPY,^[^ ^37^ ^]^ PEDOT:PSS,^[^ ^26^ ^]^ Mxene‐Graphene,^[^ ^28^ ^]^ Graphene‐AC,^[^ ^36^ ^]^ Zn//PANI‐rGO,^[^ ^29^ ^]^ MnO_2,_^[^ ^30^ ^]^ and micro‐capacitors: Zn//AC.^[^ ^27^ ^]^

Furthermore, we evaluated the areal energy of our micro‐batteries under different areal currents. At 50 µA cm^−2^, the calculated areal energies for PANI//Zn, PANI//NZn(20), PANI//NZn(40), and PANI//NZn(60) were 13.42, 13.94, 12.86, and 17.22 µWh cm^−2^, respectively. At a higher areal current of 1 mA cm^−2^, these values were 8.02, 9.86, 8.61, and 12.17 µWh cm^−2^. To further assess the performance of our PANI//NZn micro‐battery and benchmark it against previously reported micro‐scale energy storage devices, we refer to the Ragone plot presented in **Figure** **7**. At the same time, our PANI//NZn micro‐batteries exhibited impressive charge storage performance, delivering areal powers (at areal energies) of 6.93 mW cm^−2^ (at 7.69 µWh cm^−2^), 6.15 mW cm^−2^ (at 6.83 µWh cm^−2^), and 6.98 mW cm^−2^ (at 9.69 µWh cm^−2^), respectively, while that of PANI//Zn micro‐batteries was only 5.5 mW cm^−2^ (at 6.11 µWh cm^−2^). The introduction of 3D Ni scaffolds significantly enhanced the areal power density of the ZIMBs, outperforming most previously reported high‐performance polymer‐based planar micro‐batteries. For example, the Zn//PANI micro‐battery delivers a peak energy density of 5.84 µWh cm^−2^ and a peak power density of 1.86 mW cm^−2^.^[^ ^23^ ^]^ In comparison, the Zn//PANI‐GO micro‐battery reaches 2.52 µWh cm^−2^ and 0.07 mW cm^−2^.^[^ ^24^ ^]^ Although the Zn//PANI@Si battery achieves a higher energy density of 21.0 µWh cm^−2^, its power density is limited to 0.1 mW cm^−2^.^[^ ^25^ ^]^ Additional data presented in the Ragone plot further highlights the superior efficiency of our PANI//NZn micro‐batteries.^[^ ^26^, ^27^, ^28^, ^29^, ^30^, ^31^, ^32^, ^33^, ^34^, ^35^, ^36^, ^37^ ^]^ Notably, their areal power density rivals or even exceeds that of planar micro‐supercapacitors, underscoring the excellent overall performance of our design.

## 3 Conclusion

In this study, we presented an effective strategy for incorporating a 3D porous scaffold to electrodeposit Zn anodes, enabling the realization of high‐performance planar Zn‐ion micro‐batteries. Through systematic investigations of various micro‐anode configurations ‐ Zn on planar Au IDEs, porous Zn on Au IDEs, and Zn on 3D porous Ni scaffolds (NZn) ‐ we demonstrated that NZn anodes not only preserve their structural integrity but also offer superior long‐term cycling stability and effectively suppress dendrite formation. When coupled with a PANI cathode, the resulting micro‐battery achieves an impressive areal energy of 17.22 µWh cm^−2^ and an areal power of 6.98 mW cm^−2^, with a rate performance recovery exceeding 99%. Post‐cycling SEM analysis further confirmed enhanced morphological stability and dendrite mitigation in the NZn configuration. This work underscores the critical role of anode design ‐ particularly for metal‐based micro‐batteries ‐ in dictating device stability and performance and offers a promising pathway toward the development of safe, reliable, and high‐performance Zn‐ion micro‐batteries for next‐generation on‐chip energy storage applications.

## 4 Experimental Section

### Materials

Aniline (Sigma–Aldrich), Sulfric Acid(H_2_SO_4_, Fisher Bioreagents), Sodium Sulfate Anhydrous(Na_2_SO_4_, Fisher Bioreagents), Zinc Sulfate Heptahydrate (ZnSO_4_·7H_2_O, Thermo Scientific), Boric Acid (Sigma–Aldrich), Sodium Bromide (NaBr, Sigma‐Aldrich), Ammonium Acetate (Source Chemicals), Zinc Acetate Dihydrate (Sigma‐Aldrich), Nickel Chloride (NiCl_2_•6H_2_O, Scientific Laboratory Supplies), Ammonium Chloride(NH_4_Cl, Fluoro Chem), Polyvinyl alcohol (PVA, Sigma‐Aldrich), Zinc Trifluoromethanesulfonate(Zn(CF_3_SO_3_)_2_, Fluoro Chem).

### Preparation of Polyaniline (PANI) Cathode and Zn Anode

Aniline was added after the H_2_SO_4_ solution was diluted to 1 m to prepare the electrolyte of polyaniline. A mixture of Na_2_SO_4_ (12.5 g), ZnSO_4_·7H_2_O (22.3 g), and boric acid (2 g) was dissolved in deionized water (91 mL) to serve as the electrolyte for Zn deposition.

A commercial gold integrated pattern chip was held with a platinum electrode clip. The electrodeposition processes were conducted using a three‐electrode system, with an Ag/AgCl aqueous electrode as the reference electrode and a platinum wire electrode as the counter electrode. PANI was deposited using a constant voltage process at 0.85 V for 30 s. Subsequently, Zn electrochemical deposition was performed at −40 mA for 8 s.

### Preparation of 3D Zn Anode

NaBr (3 m), 1 m ammonium acetate, and 0.01 m zinc acetate were mixed in DI water to prepare the electrolyte for 3D electrodeposition. The electrodeposition was processed under a three‐electrode system using a Calomel electrode and a platinum plate as the reference electrode and the counter electrode, respectively. A constant current of −0.04 A was applied during the deposition process for 120 s to form the 3D Zn electrode.

### Preparation of 3D Ni Zn (NZn) Anode

A solution of 0.2 m NiCl_2_•6H_2_O and 2 m NH_4_Cl was prepared as the electrodeposition solution of 3D Ni Scaffold. The 3D Ni scaffold was prepared through electrodeposition in a two‐electrode system at a constant current of −2.5 A cm^−2^, while a Ni foam was used as the counter electrode. The deposition lasts for various time periods to compare the performance and effect of different thicknesses. Afterward, Zn was deposited for different time periods on the Ni scaffold as mentioned previously.

### Preparation of PVA Gel Electrolyte

PVA powder (1 g) was dissolved in deionized water (10 mL) at 85 °C. Gradually, Zn(CF_3_SO_3_)_2_ (10.9 g) was added to the solution.

## Conflict of Interest

The authors declare no conflict of interest.

## Supporting information



Supporting Information

## Data Availability

The data that support the findings of this study are available from the corresponding author upon reasonable request.
