# Supplementary material for: Enhancing Stability and Capacity in Planar Zn‐Ion Micro‐Batteries via 3D Porous Ni Anode Integration
Source: Small Methods. 2025 Sep 3;9(11):e01194. doi: 10.1002/smtd.202501194 (PMC12641344; doi:10.1002/smtd.202501194)
Supplement: Supplementary file 1 — Supporting Information [file SMTD-9-e01194-s001.pdf]

Supporting Information

for

Enhancing Stability and Capacity in Planar Zn-Ion Micro-Batteries via 3D Porous Ni Anode Integration

Yijia Zhu,[1] Xiaopeng Liu[1], Nibagani Naresh[1], Jingli Luo[1], Xueqing Hu[1], Sijin Liu,[1] Georgios Nikiforidis,[1] Mingqing Wang,[1] Buddha Deka Boruah[*,1]

[1]Institute for Materials Discovery, University College London, London WC1E 7JE, UK

*Corresponding Author:

Dr. Buddha Deka Boruah, E-mail: b.boruah@ucl.ac.uk

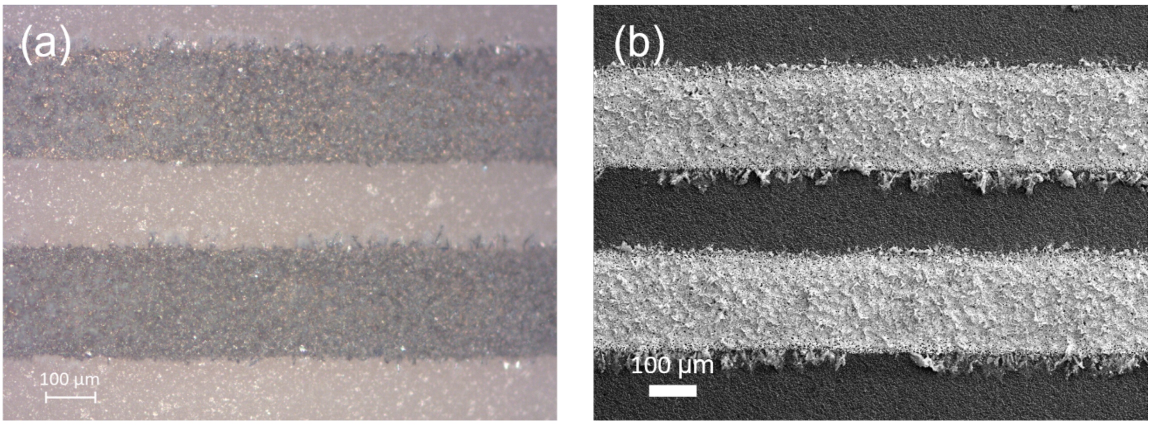

Figure S1. *a) Optical microscope image and b) SEM image of 3D Zn symmetrical cells after discharging and charging for 1 hour.*

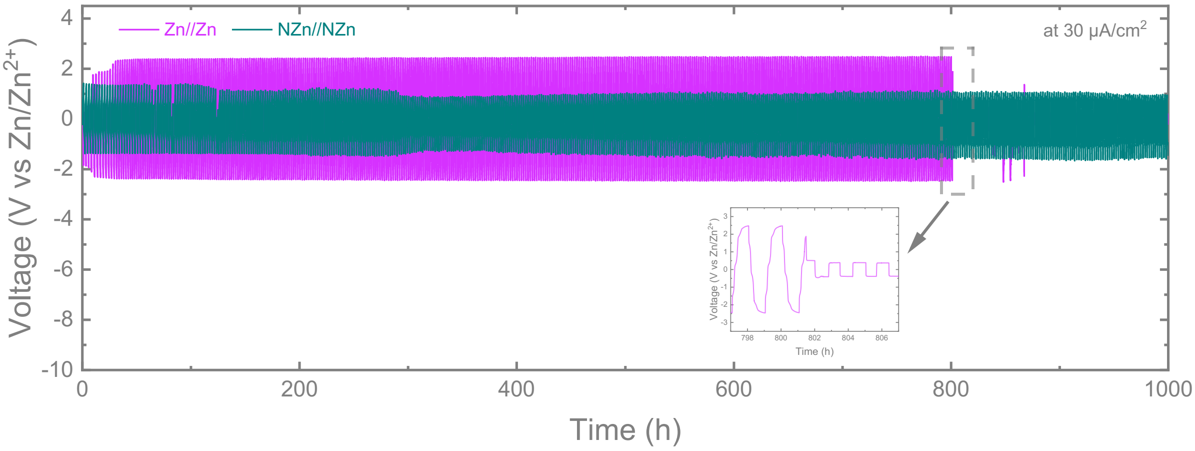

Figure S2. *Symmetrical long-term cycling curves of Zn//Zn and NZn//NZn cells.*

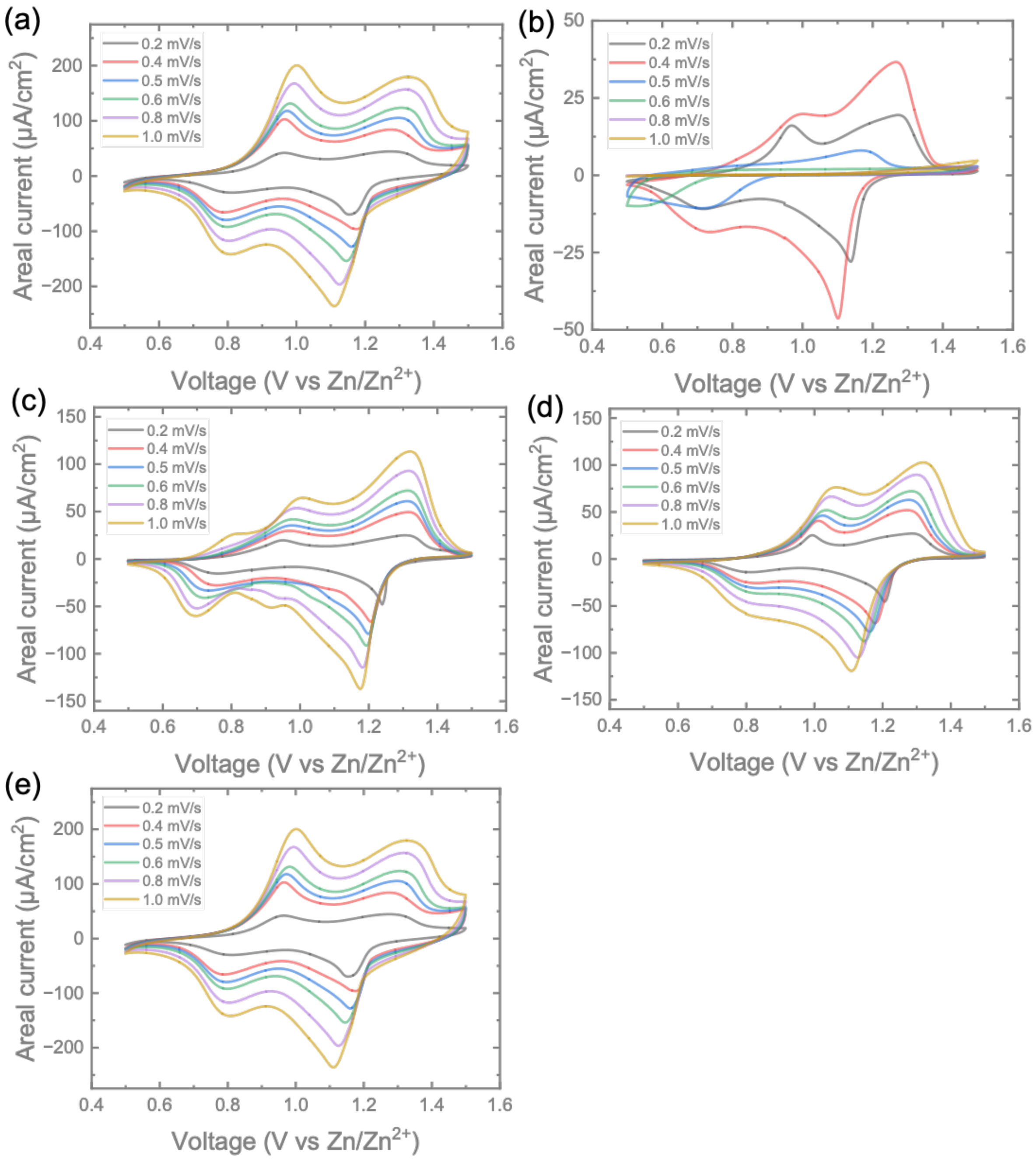

Figure S3. *CV curves of (a) PANI//Zn, (b) PANI//3D Zn, (c) PANI//NZn(20), (d) PANI//NZn(40), (e) PANI//NZn(60) micro-batteries at different scan rates of 0.2, 0.4, 0.5, 0.6, 0.8, and 1.0 mV/s.*

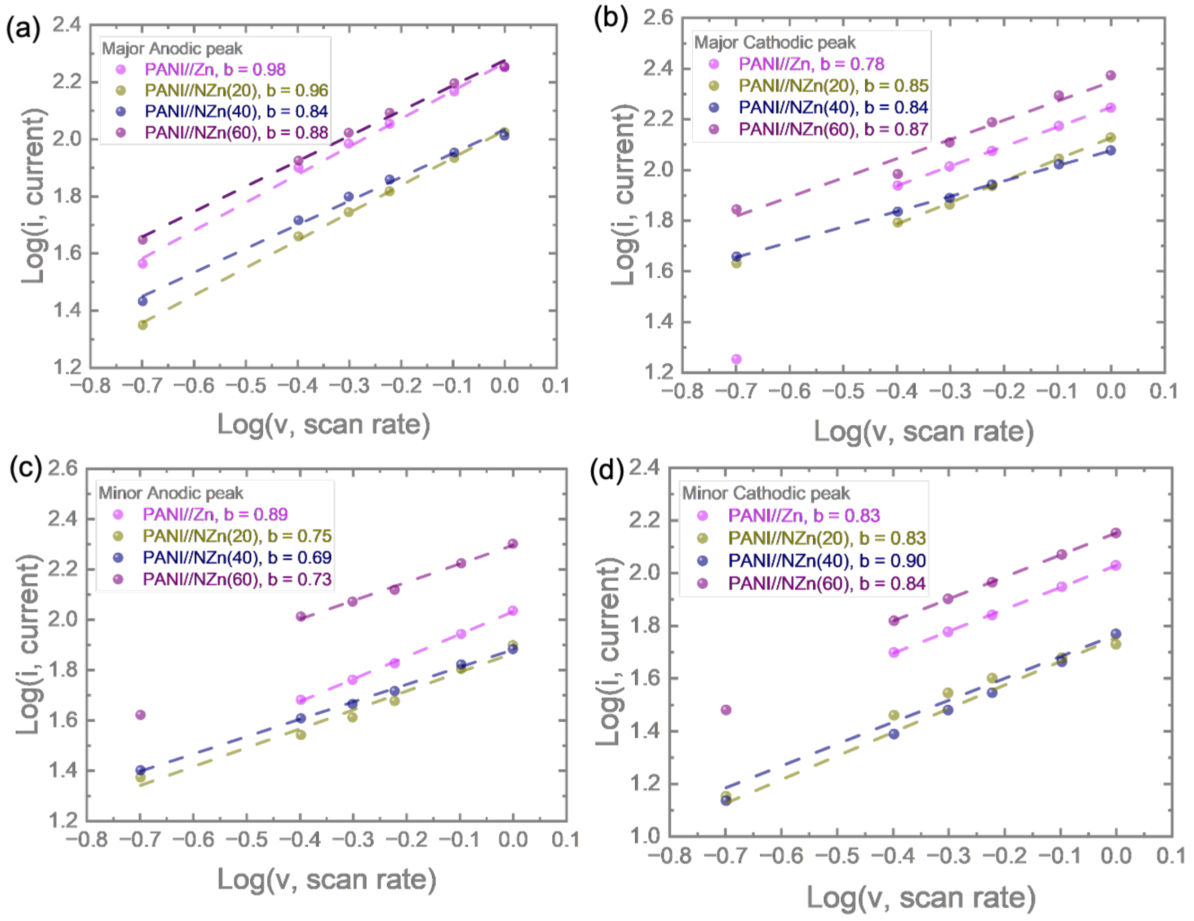

Figure S4. *b values of (a) major anodic, (b) major cathodic, (c) minor anodic, and (d) minor cathodic peaks.*

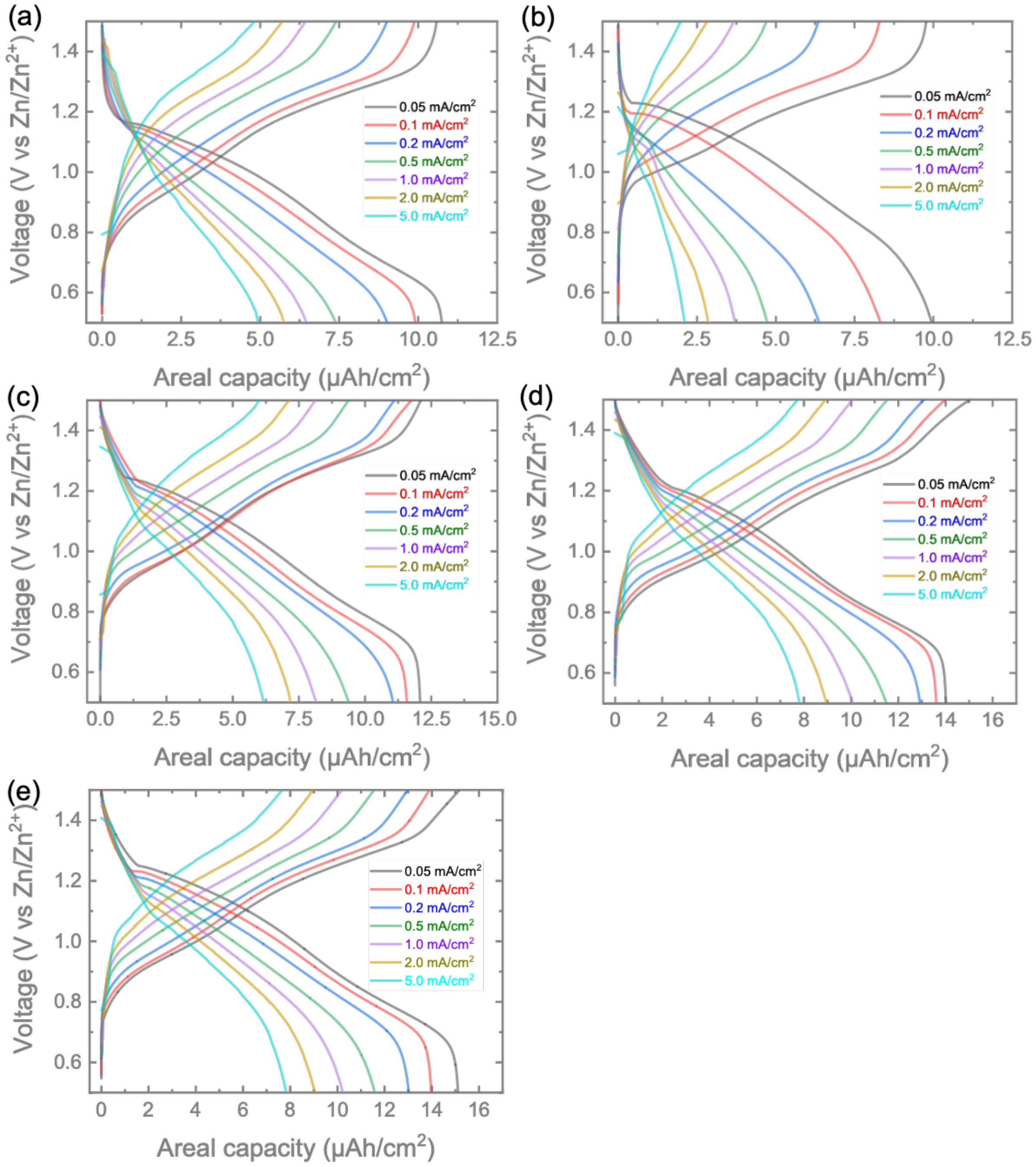

Figure S5. *GCD curves of (a) PANI//Zn, (b) PANI//3D Zn, (c) PANI//NZn(20), (d) PANI//NZn(40), (e) PANI//NZn(60) micro-batteries at different areal currents of 0.05, 0.1, 0.2, 0.5, 1.0, 2.0, and 5.0 mA cm*$^{-2}$.

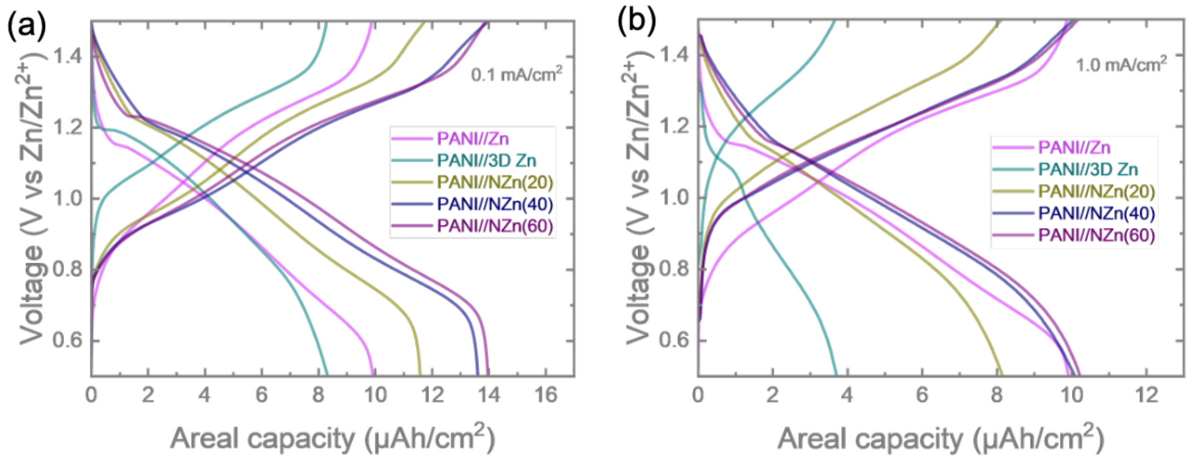

Figure S6. *Comparative GCD curves of PANI//Zn, PANI//3D Zn, PANI//NZn(20), PANI//NZn(40), PANI//NZn(60) micro-batteries at the areal currents of (a) 0.1 mA cm*$^{-2}$ *and (b) 1.0 mA cm*$^{-2}$.

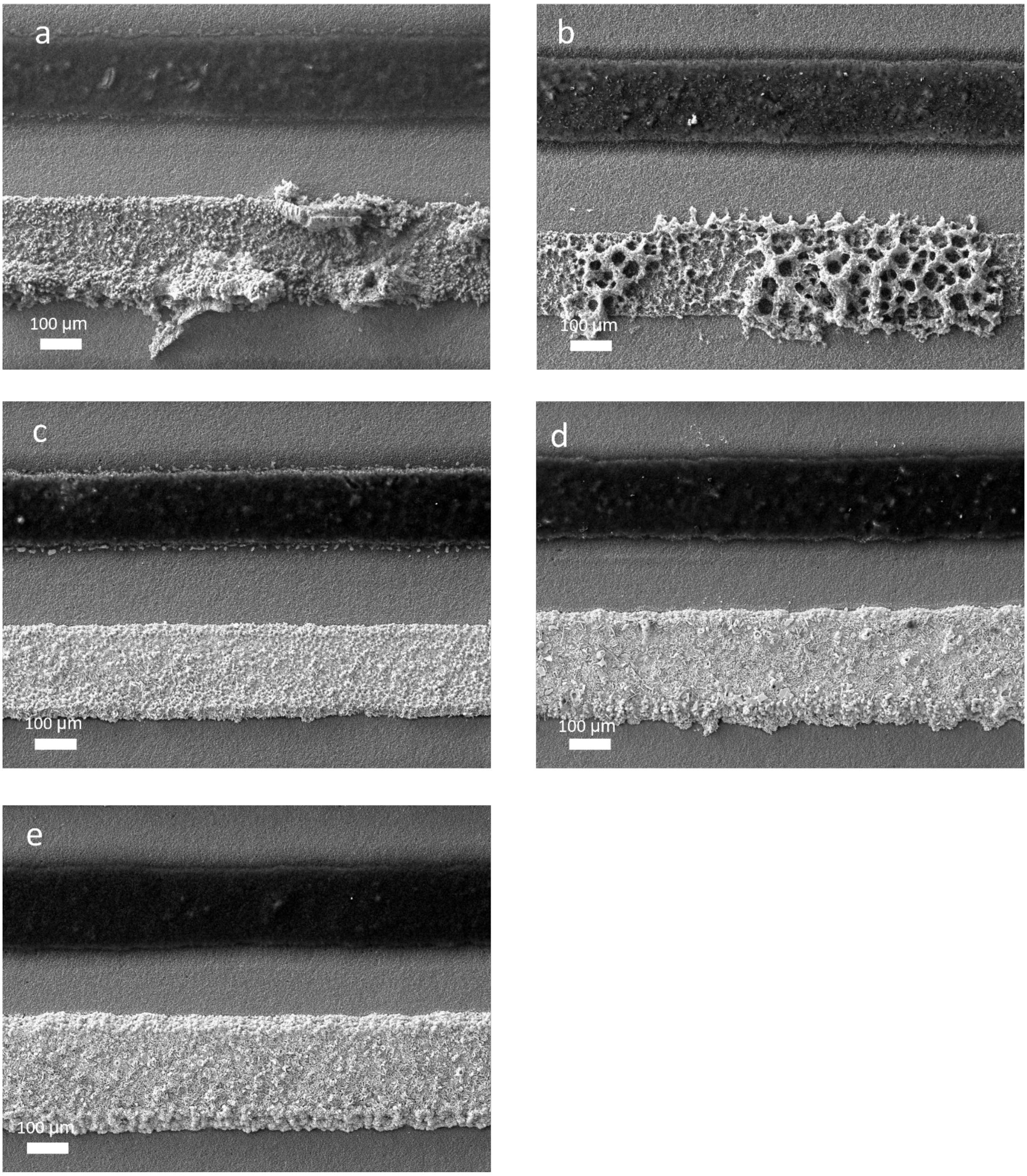

Figure S7. *Low magnification SEM images of (a) PANI//Zn, (b) PANI//3D Zn, (c) PANI//NZn(20), (d) PANI//NZn(40), and (e) PANI//NZn(60) micro-batteries after 1000 cycles.*

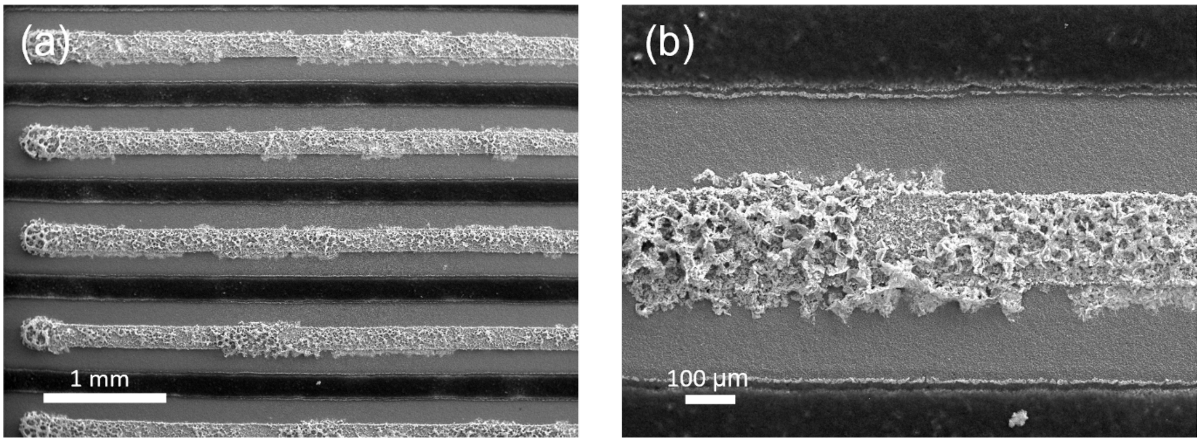

Figure S8. *SEM images of PANI//3D Zn micro-batteries after 500 cycles at a) low and b) high magnifications.*

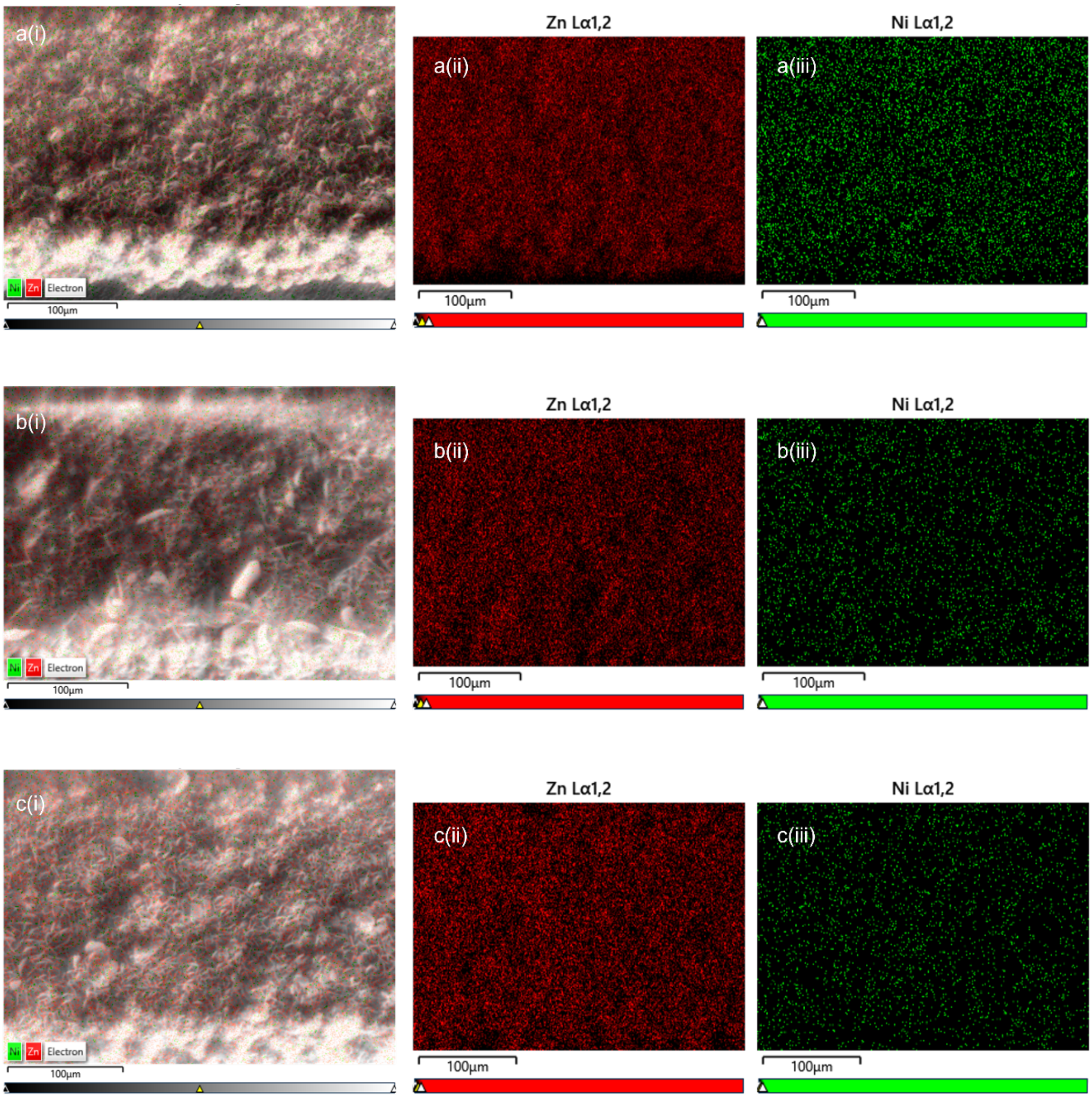

Figure S9. *EDS results exploring Zn element (ii) and Ni element (iii) on the anodes of (a) PANI//NZn(20), (b) PANI//NZn(40), and (c) PANI//NZn(60) after cycling,*
